# Botulinum toxin antibody titres: measurement, interpretation, and practical recommendations

**DOI:** 10.1007/s00415-022-11424-0

**Published:** 2022-11-24

**Authors:** Dirk Dressler, John C. Rothwell, Kailash Bhatia, Bruno Kopp, Hans Bigalke, Fereshte Adib Saberi

**Affiliations:** 1 Movement Disorders Section, Department of Neurology, Hannover Medical School, Carl-Neuberg-Str. 1, 30625 Hanover, Germany; 2 UCL Queen Square Institute of Neurology, London, UK; 3 Department of Clinical and Movement Neurosciences, UCL Queen Square Institute of Neurology, NHNN Queen Square, London, UK; 4 Department of Neurology, Hannover Medical School, Hanover, Germany; 5 Toxogen, Hanover, Germany; 6 IAB-Interdisciplinary Working Group for Movement Disorders, Hamburg, Germany; 7 Movement Disorders Section, Department of Neurology, Hannover Medical School, Hanover, Germany

**Keywords:** Botulinum toxin, Therapy, Therapy failure, Antibodies, Titres, Mouse lethality assay, Mouse diaphragm assay, Sternocleidomastoid test

## Abstract

Botulinum toxin (BT) therapy may be blocked by antibodies (BT-AB) resulting in BT-AB induced therapy failure (ABF). BT-AB may be detected by the mouse lethality assay (MLA), the mouse diaphragm assay (MDA) and the sternocleidomastoid test (SCMT). For the first time, we wanted to compare all three BT-AB tests and correlate them to subjective complaint of complete or partial secondary therapy failure in 37 patients with cervical dystonia (25 females, 12 males, age 51.2 ± 11.4 years, disease duration 12.4 ± 6.3 years). Complaint of therapy failure was not correlated with any of the BT-AB tests. MDA and MLA are closely correlated, indicating that the MDA might replace the MLA as the current gold standard for BT-AB measurement. The SCMT is closely correlated with MDA and MLA confirming that BT-AB titres and BT's paretic effect are in a functional balance: low BT-AB titres are reducing BT's paretic effect only marginally, whereas high BT-AB titres may completely block it. When therapy failure is classified as secondary and permanent, BT-AB evaluation is recommended and any BT-AB test may be applied. For MDA > 10 mU/ml, MLA > 3 and SCMT < 25%, ABF is highly likely. MDA < 0.6 mU/ml are therapeutically irrelevant. They are neither correlated with pathologic MLA nor with pathologic SCMT. They should not be the basis for treatment decisions, such as switching dystonia therapy to deep brain stimulation. All other results are intermediate results. Their interactions with therapy efficacy is unpredictable. In these cases, BT-AB tests should be repeated or one or two additional test methods should be applied.

## Introduction

Botulinum toxin (BT) therapy is a novel therapeutic principle, which expanded during the last 40 years into the treatment of numerous disorders based on muscle and exocrine gland hyperactivity [4]. More recently, chronic migraine became another indication. As predicted from the very beginning, BT's biological actions may be blocked by antibodies (BT-AB) [5] resulting in therapy failure (ABF). Dressler [2] BT-AB can be detected by different methods. Structural BT-AB tests, such as ELISA tests [10], are easy to perform, but are unable to distinguish between neutralising and non-neutralising BT-AB. Functional tests are based on BT's blockade of certain organ functions. When BT-AB are present, the normal BT effect is reduced. Functional tests can be performed in animals and humans. In animals, the mouse lethality assay (MLA) [14] monitors the overall functioning, i.e. the survival, of the mouse organism. BT induced blockade results in death, caused by respiratory and autonomic failure and probably other unspecific factors. Presence of BT-AB increases the survival rate of the mouse population. Although still considered the current gold standard, the MLA exposes the test animals to prolonged suffering and agony. An alternative animal test, the mouse diaphragm assay (MDA) [11], uses the contraction force of an explanted mouse diaphragm as test parameter. This ex-vivo test avoids animal suffering. In humans, several functional tests have been suggested. The extensor digitorum brevis test [13] monitors the BT induced paresis of the extensor digitorum brevis muscle. The sternocleidomastoid test (SCMT) [7, 8] monitors the BT induced paresis of the sternocleidomastoid muscle. Unlike the extensor digitorum brevis test, the SCMT's test methodology is based on dose–effect curves and produces quantitative results.

In this study, we wanted (1) to compare qualitatively the results of all three BT-AB tests, (2) to compare quantitatively the results of the SCMT with the results of the MDA and the MLA in order to interpret the functional relevance of the BT-AB titres measured, (3) to objectify the patients' subjective assessment of the therapy efficacy (TE) with the results of the BT-AB tests applied.

## Methods

### Design

The study is a non-interventional retrospective chart review of diagnostic data originating from routine BT treatment of patients with cervical dystonia (CD) and therapy failure (TF). Study parameters included SCMT, MDA, MLA and TE data.

### Data base

Study data originated from patients receiving BT therapy for CD in the BT outpatient clinics of the Department of Neurology, Institute of Neurology, Queen Square, London and receiving special TF workup in the Medical Research Council Human Movement and Balance Unit of the Institute of Neurology, Queen Square, London.

### Patients

Patients were included in the study, when they fulfilled the following criteria: (1) BT therapy for at least 1 year at our institution with initially satisfactory results (2) complaint of reduced or absent TE on at least three subsequent BT applications. According to the Dressler criteria [3], TF was, therefore, classified as secondary, subjective, definite, and complete or partial. Patients with these criteria were consecutively included in the study. Exclusion criteria included the inability or unwillingness to comply with the TF workup procedures.

### Sternocleidomastoid test (SCMT)

The SCMT investigates the BT induced reduction of the maximal voluntary surface electromyographic activity (M-EMG) of the sternocleidomastoid muscle [7, 8]. It compares the M-EMG reduction in a patient with suspected ABF with a control group. To perform the SCMT, the patient sitting in an upright position was asked to produce ten maximal voluntary activations of both sternocleidomastoid muscles lasting for 5 s each by performing maximal isometric head flexions from the neutral position against the resistance of the examiner. After careful cleaning the skin with acetone, M-EMG signals were recorded with two 0.5 cm diameter Ag/AgCl silver cup electrodes placed half way between the origin and the insertion of the sternocleidomastoid muscle and 3 cm distally towards the sternum. In each patient, M-EMG measurements were performed unilaterally. The electromyographic signals were filtered with offset frequencies of 10 Hz and 10 kHz and processed with a commercially available electromyography device (Nihon Koden Mini Pack Four). M-EMG amplitudes were measured within an envelope curve. After discarding the two maximal voluntary activations with the lowest amplitudes, the remaining eight were used to calculate the M-EMG. Co-operation of the test person to perform maximal head flexions was tested by application of different counterforces and by comparison with contralateral head rotations. The M-EMG reduction was determined by comparing the pre-BT and the post BT M-EMG measured 14–21 days after BT application. The reduction was expressed as a percentage of the pre-BT M-EMG.

All BT applications were performed with abobotulinumtoxinA (Dysport^®^, Ipsen Ltd, Maidenhead, Berks, UK). 500 Ipsen mouse units (MU-I) were reconstituted with 2.5 ml 0.9% NaCl/H_2_0 and applied within 30 min after reconstitution. Storage and handling conditions as described by the manufacturer were carefully observed. The BT dose used for this test was 300MU-I. It was divided into three equal portions and applied with a 0.45 × 12 mm needle into three injection sites distributed equally over the sternocleidomastoid muscle belly. After the BT application, the injection sites were compressed for 1 min to avoid haematoma and to improve BT tissue penetration.

The M-EMG reduction was determined as pathological, when its value was more than two standard deviations below the mean previously measured in a control group, i.e. less than 66%. Results are given as SCMT-XX%.

### Mouse diaphragm assay (MDA)

To perform the MDA [11], the left phrenic nerve together with the left hemidiaphragm were excised from male or female Naval Medical Research Institute (NMRI) mice of 18–22 g body weights and placed in an organ bath containing 3.5 ml of Krebs–Ringer solution composed of 118 mM NaCl, 4.75 mM KCl, 2.54 mM CaCl_2_, 1.19 mM KH_2_PO_4_, 1.2 mM MgSO_4_, 1.2 mM NaHCO_3_, 11 mM glucose and 0.1% bovine serum albumin. The pH value was adjusted to 7.4 by gassing with 95% O_2_ and 5% CO_2_. The phrenic nerve was then continuously electro-stimulated at a frequency of 1 Hz via 2 ring electrodes. The electrostimulation was performed using a pulse duration of 0.1 ms and a supramaximal amplitude of 3 mV. Isometric contractions of the hemidiaphragm were recorded with a force transducer (Scaime, France) and a pen recorder (Hellige, Germany). The resting tension of the hemidiaphragm preparation was approximately 15 mN. Indirectly stimulated control muscles maintained an undiminished contractile response (twitch) for periods of more than 4 h. In each experiment, the phrenic nerve-hemidiaphragm preparation was first allowed to equilibrate for 15 min under controlled conditions. Then, the incubation medium was exchanged for the BT containing solution. BT concentrations were set to 1 ng/ml for the purified neurotoxin and to 2 ng/ml for the complex to allow reduction of the twitch amplitude by 50% within 3 h. After BT application, the twitch amplitude remained unchanged for some time before it slowly decreased with a velocity correlated with the BT concentration. The time required for reduction of the twitch amplitude by 50% was named paralysis time and used as test parameter.

For determination of BT-AB titres a specific anti-botulinum A toxin F(ab')2 serum (equine Fermo serum, Behring-Werke, Germany) containing 750 U/ml was used as a standard to construct a calibration curve after taking the dilution factor into account. BT had a LD50 of 35 pg and was obtained from Dr E Schantz, Madison, WI, USA.

The MDA was deemed negative, when the paralysis time of the test sample was within two standard deviations of the mean paralysis time of the control population, i.e. 66 min. AB titres > 0.6 mU/ml was classified as positive. AB readings > 10 mU/ml were reported as 10 mU/ml. Results are given as MDA-XX.XmU/ml.

### Mouse Lethality assay (MLA)

The MLA investigates whether a patient serum contains AB exerting a protective effect against a controlled lethal intoxication of a specially bred mouse population. The MLA used for this study was similar to MLA previously reported [14]. It was provided by Ipsen Ltd, Maidenhead, Berks, UK. Further details about this MLA were not available. Results of AB levels were reported as negative (0), low-positive (1), low/intermediate-positive (2), intermediate-positive (3), intermediate/high-positive (4) and high-positive (5). Data on linearity of the result scale were not available. Results are given as MLA-X.

### Therapy effect (TE)

TE was classified as partial secondary therapy failure (PSTF), when therapeutic efficacy and adverse effects (if initially present) were reduced on three subsequent BT injection series after they had been acceptable at therapy initiation. Complete secondary therapy failure (CSTF) was diagnosed, when there was no therapeutic efficacy and no adverse effects (if initially present) on three subsequent BT injection series. Classification was based on the patient's subjective judgement.

### Statistics

The statistical significance level was set to *p* = 0.05. The statistical tests applied are indicated in the text.

## Results

### Isolated results

Altogether, 37 patients (25 females, 12 males, age at evaluation 51.2 ± 11.4 years, duration of CD at evaluation 12.4 ± 6.3 years) were studied. TE was PSTF in 20 patients and CSTF in 17 patients. SCMT was pathological in 17 patients and normal in 20 patients. Pathological values ranged from SCMT-2% to SCMT-58%. MDA was pathological in 16 patients and normal in 21 patients. Pathological values ranged from MDA-0.6 mU/ml to MDA-10.0 mU/ml. MLA was pathological in 10 patients. Values were 5 in 6 patients, 4 in 2 patients and 1 in 2 patients. 27 patients showed normal results.

### Qualitative correlations

**SCMT/MDA correlation**: In 32 of all 37 patients tested, results were congruent in the SCMT and the MDA. In 14 of them, both tests were pathological and in 18, both tests were normal. In five patients, the results were incongruent. Pearson's chi-squared test revealed that the null hypothesis of independently distributed SCMT and MDA should be rejected (*Χ*^2^ (*df* = 1) = 19.600; *n* = 37; *p* < 0.001), i.e. that SCMT and MDA were significantly correlated.

**SCMT/MLA correlation**: In 33 of all 37 patients tested, results were congruent in the SCMT and the MLA. In 14 of them, both tests were pathological and in 19, both tests were normal. In 4 patients, results were incongruent. Pearson's chi-squared test revealed that the null hypothesis of independently distributed SCMT and MLA should be rejected (*Χ*^2^ (*df* = 1) = 16.122; *n* = 37; *p* < 0.001), i.e. that SCMT and MLA were significantly correlated.

**MDA/MLA correlation**: In 31 of all 37 patients tested, results were congruent in the MDA and the MLA. In 10 of them, both tests were pathological and in 21, both tests were normal. In six patients, results were incongruent. Pearson's chi-squared test revealed that the null hypothesis of independently distributed MDA and MLA should be rejected (*Χ*^2^ (*df* = 1) = 17.986; *n* = 37; *p* < 0.001), i.e. that MDA and MLA were significantly correlated.

**MDA/TE correlation**: In 14 of all 37 patients tested, normal MDA was correlated with PSTF, in 7 with CSTF. In 10 of all 37 patients tested, pathological MDA was correlated with CSTF, in 6 with PSTF. Pearson's chi-squared test revealed that the null hypothesis of independently distributed MDA and TE cannot be rejected (*Χ*^2^ (*df* = 1) = 3.111; *n* = 37; *p* = 0.078), i.e. that MDA and TE were not correlated.

**MLA/TE correlation**: In 17 of all 37 patients tested, normal MLA was correlated with PSTF, in 10 with CSTF. In 7 of all 37 patients tested, pathological MLA was correlated with CSTF, in 3 with PSTF. Pearson's chi-squared test revealed that the null hypothesis of independently distributed MLA and TE cannot be rejected (*Χ*^2^ (*df* = 1) = 3.193; *n* = 37; *p* = 0.074), i.e. that MLA and TE were not correlated.

**SCMT/TE correlation**: In 13 of all 37 patients tested, normal SCMT was correlated with PSTF, in 7 with CSTF. In 10 of all 37 patients tested, pathological SCMT was correlated with CSTF, in 7 with PSTF. Pearson's chi-squared test revealed that the null hypothesis of independently distributed SCMT and TE cannot be rejected (*Χ*^2^ (*df* = 1) = 2.100; *n* = 37; *p* = 0.147), i.e. that SCMTT and TE were not correlated.

**SCMT/MDA/MLA correlation**: In 28 of all 37 patients tested, all three methods generated congruent results. In 18 of them, all methods produced normal results, in ten pathological results. In nine patients, results were incongruent. In all of those patients, the MLA was normal, whereas in four of them, the SCMT and the MDA were pathological. In the remaining 6 patients, either the SCMT or the MDA were pathological.

**SCMT/MDA/MLA/TE correlation**: In all 18 patients with normal results in all three methods, 12 patients showed PSTF and six CSTF. In all ten patients with pathological results in all three methods, three patients showed PSTF and 7 CSTF.

### Quantitative correlations

**Fig. 1:**
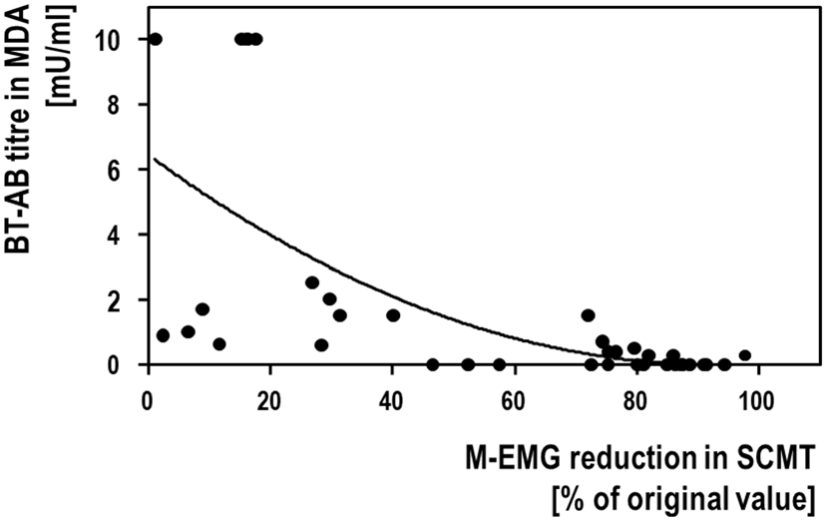
Correlation between the results of the mouse diaphragm assay (MDA) and the sternocleidomastoid test (SCMT). Both parameters were significantly correlated with each other (see text)

**SCMT/MDA correlation**: Figure 1 shows the quantitative correlation between the SCMT and the MDA. Spearman's rank correlation coefficient (rho) amounted to *ρ* = − 0.764 (*n* = 37; *p* < 0.001), i.e. both parameters were significantly correlated.

**Fig. 2:**
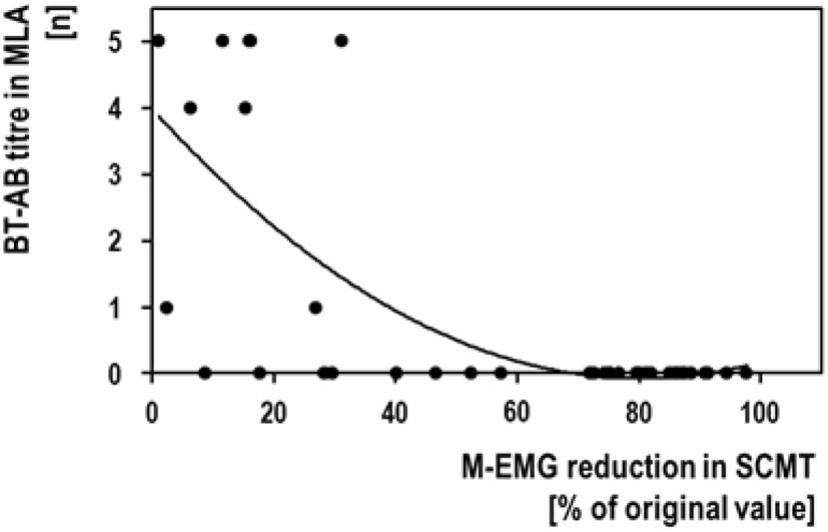
Correlation between the results of the mouse lethality assay (MLA) and the sternocleidomastoid test (SCMT). Both parameters were significantly correlated with each other (see text)

**SCMT/MLA correlation**: Figure 2 shows the quantitative correlation between the SCMT and the MLA. Spearman's rank correlation coefficient (rho) amounted to *ρ* = − 0.703 (*n* = 37; *p* < 0.001), i.e. both parameters were significantly correlated.

**Fig. 3:**
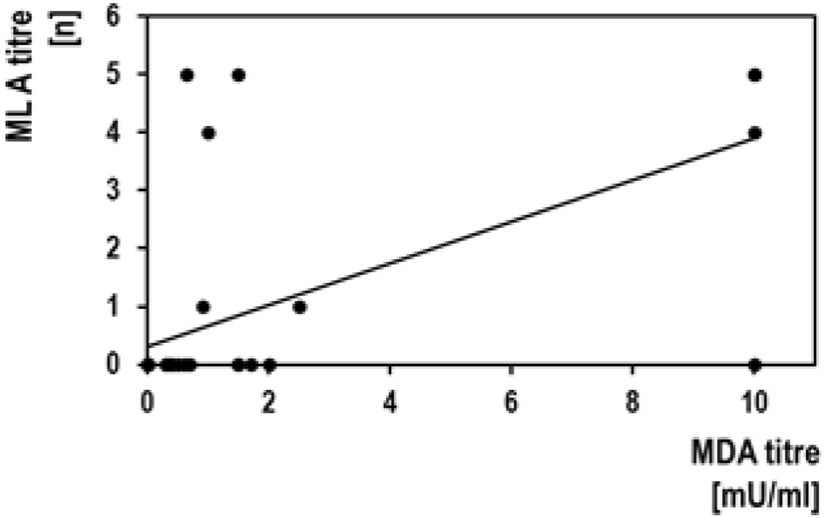
Correlation between the results of the mouse lethality assay (MLA) and the mouse diaphragm assay (MDA). Both parameters were significantly correlated with each other (see text)

**MDA/MLA correlation**: Figure 3 shows the quantitative correlation between the MDA and the MLA. Spearman's rank correlation coefficient (rho) amounted to *ρ* =  + 0.708 (*n* = 37; *p* < 0.001), i.e. both parameters were significantly correlated.

**MDA/MLA/SCMT correlation**: MDA < 0.6 mU/ml are neither correlated with pathological MLA nor with pathological SCMT. All five patients with MDA-10 mU/ml were correlated with congruent pathological MLA and SCMT. Of the 11 patients with MDA-0.6 mU/ml to MDA-10 mU/ml, 5 patients showed congruent pathological results and 6 had incongruent SCMT and MLA.

**Fig. 4:**
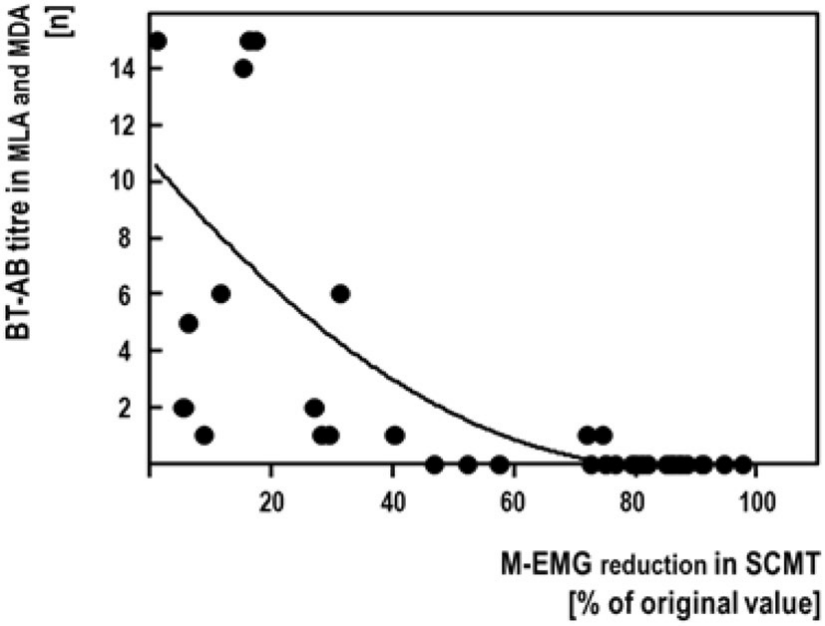
Correlation between the results of the sternocleidomastoid test (SCMT) and the combined results of the mouse diaphragm assay (MDA) and the mouse lethality (MLA) assay. Results of the MDA had been transformed into categorical values of 0–5

**SCMT/MDA + MLA correlation**: Figure 4 shows the quantitative correlation between the SCMT and the combined results of the MDA and the MLA. For this, the MDA titres had been transformed into categorical values of 0–5. Spearman's rank correlation coefficient (rho) amounted to *ρ* = − 0.836 (*n* = 37; *p* < 0.001).

## Discussion

### General

To our knowledge, this is the only study comparing several diagnostic tools to measure BT-AB titres in TF patients. Also for the first time, this study evaluates the relevance of BT-AB titres for the patient's muscle response.

### Patients

The sample of CD patients studied present with typical age of onset and typical female preponderance previously described [6].

### TE

About half of the patients complaint of PSTF and half of CSTF. Complaints of PSTF or CSTF were not correlated with any of the other study parameters. This means, that PSTF and CSTF does not allow a prediction about the presence or absence of BT-AB or ABF. This is important to realise, as many studies on BT-AB and ABF are using TE as a surrogate marker for the presence of BT-AB [12].

### MDA/MLA comparison

On a qualitative as well as on a quantitative level, results of the MDA and the MLA are closely correlated (Fig. 3) confirming previous studies [7, 8]. This means, that the MDA has the potential to replace the MLA as the current gold standard for BT-AB measurement supporting the international move to reduce animal consumption for BT drug manufacturing [1]. Trilateral comparison between the MLA, the MDA and the SCMT suggests, that the MLA may have a slightly reduced sensitivity than the other tests. This would confirm the impression arising from the previous comparison between the MLA and the MDA [7, 8].

### SCMT/MDA/MLA comparison

Our study confirms, that the SCMT is closely correlated with the MDA and with the MLA—on a qualitative as well as on a quantitative level. When results of the MDA and the MLA are merged, this quantitative correlation is confirmed (Fig. 4). This supports the previously formulated hypothesis, that BT-AB and BT are functionally correlated [5]. This means, that low BT-AB titres are affecting BT's biological activity only marginally, whereas high BT-AB titres may completely block it. This observation explains that low BT-AB titres may be functionally irrelevant and, as such, are not interfering with BT therapy. This observation also explains the finding, that intermediate BT-AB titres may be overcome by increased BT doses [9].

### Practical considerations

BT-AB tests are used to explore ABF. Our results suggest the following recommendations:

1. Patient reports of TF are usually the starting point to investigate ABF. TF should be classified according to the Dressler classification. When TF is classified as secondary and permanent, ABF may be possible and AB evaluation is recommended. If this is not the case, ABF can be excluded. Distinction between PSTF and CSTF has no predictive value for ABF.
2. Any one of the three BT-AB tests used in this study may then be applied. The choice seems arbitrary.
3. In MDA > 10 mU/ml, MLA > 3 and SCMT < 25%, ABF is highly likely, as all other BT-AB test produced congruent pathological results in our study. Test repetitions should be performed 3–6 months later. A confirmatory test with a different methodology may or may not be considered.
4. MDA < 0.6 mU/ml are therapeutically irrelevant. They are correlated neither with pathologic MLA nor with pathologic SCMT. They cannot be the basis for decisions on the patient's further treatment such as switching dystonia therapy from BT therapy to deep brain stimulation. The interpretation of MDA results should include the category 'relevant titres' [5]. Irrelevant titres may be followed up occasionally. In our experience, they do not tend to increase and do not become relevant ones.
5. All other results are intermediate results. They include MDA-0.6 mU/ml to MDA-10 mU/ml, MLA-1 to MLA-2 and SCMT-66% to SCMT-25%. They are relatively rare confirming the hypothesis, that the organism exposed to BT either responds with BT-AB formation or not [5]. The relevance of intermediate results for the muscle response and for TE are not predictable. If intermediate results are obtained, tests should be repeated or one or two additional BT-AB test methods should be applied. BT dose escalation might be helpful in those patients to overcome TF [9]. Whether these intermediate BT-AB titres will—with or without dose escalation—eventually increase, is not clear. Application of low antigenicity BT drugs seems to be advisable, especially for those patients.

**Fig. 5:**
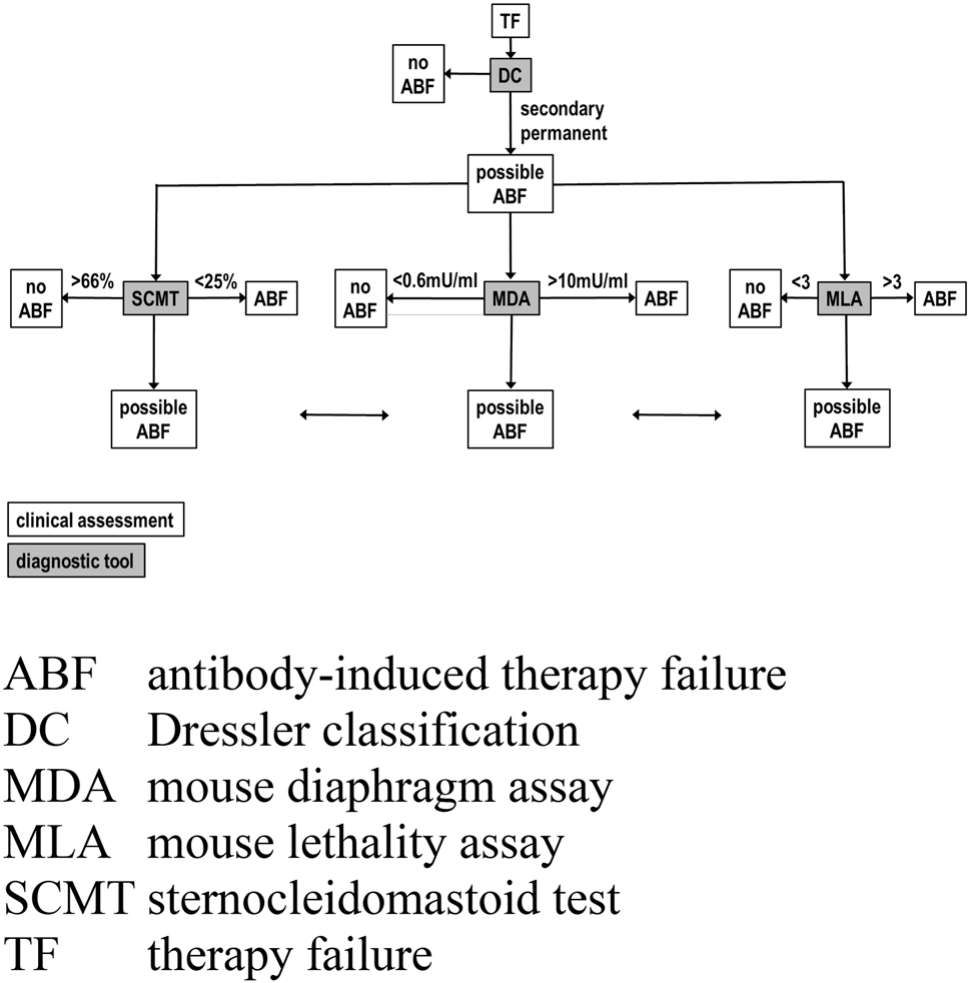
Algorithm for workup of botulinum toxin therapy failure (TF)

Figure 5 shows an algorithm for evaluation of TF. When the patient complains of TF, the Dressler classification may be used to confirm secondary permanent TF. If this is the case, diagnostic tests are applied. They include SCMT, MDA and MLA. In SCMT < 25%, MDA > 10 mU/ml and MLA > 3 ABF can be diagnosed. In SCMT < 66%, MDA < 0.6 mU/ml and MLA < 3 ABF can be excluded. All other results are intermediate. Tests should then be repeated or different test methods should be applied.

## Abbreviations

BT-AB: Antibody against botulinum toxin
ABF: Antibody-induced therapy failure
BT: Botulinum toxin
CD: Cervical dystonia
CSTF: Complete secondary therapy failure
MDA: Mouse diaphragm assay
M-EMG: Maximal voluntary surface electromyographic activity
MLA: Mouse lethality assay
MU-I: Ipsen mouse unit
PSTF: Partial secondary therapy failure
SCMT: Sternocleidomastoid test
TE: Therapy effect
TF: Therapy failure
